# Effects of Within-Person Variability in Spot Urinary Sodium Measurements on Associations With Blood Pressure and Cardiovascular Disease

**DOI:** 10.1161/HYPERTENSIONAHA.120.16549

**Published:** 2021-09-20

**Authors:** Federica Re, Imen Hammami, Thomas J. Littlejohns, Matthew Arnold, Sarah Lewington, Robert J. Clarke, Jennifer L. Carter

**Affiliations:** From the Clinical Trial Service Unit and Epidemiological Studies Unit (CTSU), Nuffield Department of Population Medicine, University of Oxford, United Kingdom (F.R., I.H., T.J.L., S.L., R.J.C., J.L.C.); British Heart Foundation Cardiovascular Epidemiology Unit, Department of Public Health and Primary Care, University of Cambridge, Cambridge, United Kingdom (M.A.); MRC Population Health Research Unit (S.L.).

**Keywords:** blood pressure, cardiovascular disease, cohort study, hypertension, sodium

## Abstract

Randomized trials of salt restriction have consistently demonstrated that decreasing salt consumption lowers blood pressure, but results of observational studies of salt intake and cardiovascular disease have been conflicting. After excluding individuals with prevalent cardiovascular or kidney disease in the prospective UK Biobank study, we examined the within-person variability in spot urinary sodium excretion and its impact on associations with systolic blood pressure and risk of incident cardiovascular disease. Spearman correlation coefficients were used to assess within-person variability in spot urinary sodium, and associations between sodium and blood pressure were assessed using linear regression in participants with measurements at baseline (N=355 134) and after 9 years (N=33 915). Cox regression was used to assess associations with the risk of cardiovascular disease over the same follow-up period (N=5566 events). The within-person variability in urinary sodium was extreme, with a self-correlation coefficient of 0.35 over 4 years. Each 100 mmol/L higher usual urinary sodium was associated with 3.09 mm Hg higher systolic blood pressure (95% CI, 2.7–3.48) at baseline, but had no association at 9 years (0.97 [−0.44 to 2.37]). Likewise, there was no association between urinary sodium and risk of cardiovascular disease over the same follow-up period (hazard ratio, 1.05, [0.87–1.26]). While spot urinary sodium measurements were associated with immediate effects on blood pressure at baseline, the extreme within-person variability in urinary sodium precluded detection of associations with future blood pressure at resurvey or risk of cardiovascular disease. The limitations of observational studies, irrespective of study size, should be recognized when assessing public policy on salt restriction.

## Abstract 2

Supplemental Digital Content is available in the text.

**See Editorial, pp 1637–1639**

Higher levels of blood pressure are associated with higher risks of stroke and ischemic heart disease, with a 20 mm Hg higher systolic blood pressure (SBP) being associated with an ≈2-fold higher risks of death from cardiovascular disease (CVD) outcomes.^1^ Elevated blood pressure accounts for almost half of all deaths from CVD worldwide.^2,3^ Randomized trials have consistently demonstrated that restriction of dietary salt intake (dietary sodium) lowers blood pressure, prompting the World Health Organization to recommend limiting dietary sodium intake to <2.3 g/d (equivalent to 6 g/d of salt).^4–6^ However, several prospective cohort studies have recently reported that intakes of dietary sodium <4 g/d were associated with higher risks of CVD mortality, and these results were used to challenge the validity of the World Health Organization’s recommendations on salt restriction for prevention of CVD.^7–10^

The reasons for the discrepant results between the beneficial associations of salt restriction on levels of blood pressure observed in randomized trials, and the apparent null or possible hazardous associations of low intakes of dietary salt with risks of CVD observed in prospective cohort studies remain unexplained.^6–9^ The aims of the present study were to use the large prospective UK Biobank study to: (1) examine the magnitude of within-person variability in spot measurements of urinary sodium (UNa); (2) assess associations of UNa with mean levels of SBP at baseline and at resurvey 9 years later, and (3) examine associations of UNa with the risk of incident CVD events during the same follow-up period.

## Methods

All results from this analysis are returned to UK Biobank within 6 months of publication, at which point they can be made available to other researchers upon reasonable request. UK Biobank is an open access resource, and researchers can apply to use the data set at http://ukbiobank.ac.uk/register-apply/.

### Study Population

This study used data from 502 619 men and women in the UK Biobank who were aged 40 to 69 years at recruitment between 2006 and 2010.^11^ Participants were invited to attend one of 22 baseline assessment centers within 25 miles of 22 major cites located throughout England, Scotland, or Wales. Data were collected on sociodemographic, lifestyle, and health-related characteristics using an electronic touchscreen questionnaire. All participants had their blood pressure and anthropometry recorded, and blood and urine samples were collected for long-term storage. Repeat measurements of all assessments, including urine samples, were collected on a sample of 20 346 participants at 4 years after baseline (2012–2013), and repeat questionnaires and blood pressure measurements were also recorded in 33 915 participants at 9 years after baseline (2016–2019). See Figure S1 in the Data Supplement for a flowchart of measurements used in the present analyses. Ethical approval was obtained from the North-West Multi-Centre Research Ethics Committee (REC reference: 11/NW/03820) and all participants provided written informed consent.

### Measurement of UNa

During both the baseline assessment and resurvey visit at 4 years after baseline, nonfasting spot urine samples were collected from all participants. Samples were transferred from the collection vessels to vacutainers and stored at 4 °C in temperature-controlled boxes before overnight shipment to the coordinating centre laboratory for long-term storage.^12^ Urine analyses were performed at 24 to 36 hours after sample collection using a Beckman Coulter AU5400.^12^

UNa excretion in spot urine samples was primarily measured as crude UNa values (mmol/L). Since spot UNa samples are confounded by diurnal variation, in addition to dilution by urine volume,^13^ measures of spot UNa were standardized to urinary creatinine concentrations using the INTERSALT equation to estimate 24-hour excretion of UNa (g/d; Methods S1). This formula takes account of spot creatinine concentrations, spot potassium excretion, age, sex, and body mass index (BMI) measurements. Hence, it should be less affected by diurnal and other sources of within-person variation.^14,15^ Since the validation of the INTERSALT equation is only moderate (which is the case for all 24-hour estimating equations), the results are reported alongside the estimates for crude UNa values; results using the Kawasaki equation of estimated 24-hour excretion (which are presented for comparison with previous research) are reported in the supplement since this equation was not validated for the type of urine sample in UK Biobank.^14,16,17^

### Measurement of Blood Pressure

Blood pressure was measured twice using an Omron HEM-7015IT digital sphygmomanometer after participants had been in the seated position for at least 5 minutes.^18^ Measurements were recorded manually by trained staff for participants in whom blood pressure could not be measured electronically. The mean levels of both measurements of SBP were used in the analysis.

### Ascertainment of Incident CVD

Incident CVD events were recorded by linkage with hospital inpatient records from the Hospital Episode Statistics for England (1996 onwards), the Scottish Morbidity Record (1981 onwards), and the Patient Episode Database for Wales (1998 onwards). Incident CVD was defined as hospital admission for a primary or secondary diagnosis of nonfatal or death from myocardial infarction or stroke (ICD-10 codes I2I-I23, 124.1, I25.2, and I60-I64 [excluding I62]). Censoring dates were 31st October 2016 for Scotland; 29th February 2016 for Wales; and 31st March 2017 for England.

### Statistical Analyses

#### Cross-Sectional Analyses

To examine the convergent validity of associations between crude UNa excretion (mmol/L) and self-reported dietary intake of foods high in sodium or potassium at baseline, sex- and age-adjusted means of crude UNa levels were compared across the frequency of adding salt to food (from never to always), consuming processed meat (from never to almost daily), and intake of fruit and vegetables (quartiles of usual daily portions). Spearman correlations were estimated between UNa and ordinal categories of food intake.

To assess within-person variability in the baseline measures of UNa, in addition to relevant covariates (Table S2), Spearman correlation coefficients between repeat measurements at baseline and at 4 years after baseline were estimated in a sample of participants with repeat measurements (N=20 346). To estimate the reproducibility of 24-hour sodium excretion independent of the reproducibility of covariates included in the INTERSALT estimating equation, repeat 24-hour UNa measurements were regressed on baseline 24-hour UNa, after adjustment for age, sex, BMI, potassium, and creatinine.

Multivariable linear regression was used to estimate cross-sectional associations of UNa with mean levels of SBP measured at baseline. The effects of possible confounding factors were assessed by comparing the differences in the *F* test values (which are reported as χ^2^ values due to their equivalence for a large population size and for ease of interpretation) for UNa across different models. The baseline model 1 adjusted for age and sex. Model 2 included adjustment for sociodemographic factors and health behaviours (see Methods S2 for details: ethnicity; family history of hypertension; season; time of day of urine sample; usual fluid consumption; education; Townsend index of deprivation; smoking; alcohol intake, physical activity; and exogenous hormones).^19–21^ Model 3 additionally adjusted for the spot urinary potassium/creatinine ratio.^21^ Finally, model 4 also adjusted for BMI (height/weight [kg/m^2^]). The same adjustments were also used when assessing associations with estimated 24-hour UNa using the INTERSALT equation.

To ensure the results of the cross-sectional and prospective associations between UNa and SBP were not due to different sample sizes, a sensitivity analysis examined the cross-sectional analyses when restricted to those that had SBP measurements at both baseline and at resurvey (N=33 915; Figure S3).

#### Prospective Analyses

The association of baseline UNa with SBP measured at a resurvey 9 years after baseline (future SBP) was estimated using multivariable linear regression with adjustment for the same confounders outlined above. Since the association of UNa with the risk of incident CVD is mediated by the association with blood pressure, the association of UNa with CVD was estimated for a similar follow-up period as those with a resurvey of SBP measurements.^22^ Cox regression was used for analyses of associations with CVD, where the follow-up was calculated until the first CVD event after recruitment, death, or otherwise censored. Models were stratified by 5-year age bands at recruitment and were adjusted for the confounders outlined above.

Since the strength of the associations of baseline UNa with SBP and CVD are likely to be weaker than the associations of usual (ie, long-term average) levels of UNa, all analyses were corrected for regression dilution using previously reported methods.^23,24^ Participants were first ranked into groups of UNa at baseline (quintiles, with the lowest quintile of UNa divided further into tertiles for more detail), and the mean values of UNa at resurvey in those baseline defined groups (ie, the usual values) were used when plotting results. Assuming the associations were linear, analyses for 100 mmol/L higher usual crude UNa (and for 1 g/d higher usual 24-hour INTERSALT UNa) were estimated by dividing the β coefficient and their standard errors by the regression dilution ratio obtained between baseline and resurvey UNa.^23^ These adjustments should correct for attenuation due to random error, but they will not affect systematic errors in measurements of UNa. Further sensitivity analyses checked the associations of SBP measured at baseline with the risk of CVD (Figure S5).

### Patient and Public Involvement

Participants were not involved in formulation of any research questions or outcome measures used in the present report, but were consulted about the ethics and governance frameworks, and engaged in periodic reviews of UK Biobank using follow-up questionnaires, additional assessment visits, participant events, and newsletters.^25^

## Results

### Baseline Characteristics

Analyses excluded participants with missing data on UNa (N=16 518), SBP (N=412), BMI (N=1227), and other covariates (N=3685). To limit the effects of reverse causality, analyses excluded participants with a history of prior CVD (N=33 301), kidney disease (N=3017), macroalbuminuria (N=1552), or diabetes (N=20 962) at baseline; CVD events occurring during the first 2 years of follow-up were also dropped (N=1044). Participants reporting use of antihypertensive medication at baseline were also excluded to limit reverse causality (N=66 811). For participants that began taking blood pressure-lowering medication after baseline (and after the measurement of UNa; N=4102; 12.1%), a constant of 10 mm Hg was added to future SBP values.^26,27^ The main analyses were based on 355 134 participants (70.7% of the original sample), including 33 915 participants who had SBP measurements recorded at both baseline and resurvey.

The mean age of the study participants was 55.2 years (SD, 8.1), 4.7% were from a non-White ethnicity, and 42.8% were men (Table 1). Mean (SD) levels at baseline of SBP were 135.9 (18.4) mm Hg and diastolic blood pressure were 81.8 (10.1) mm Hg, and the mean levels at resurvey 9 years later were 139.0 (19.2) and 78.6 (10.0) mm Hg, respectively. Mean (SD) levels of UNa were 76.4 (44.3) mmol/L and increased from an average of 26.7 (6.9) to 147.0 (29.5) mmol/L (and from 2.3 [0.2] to 4.4 [0.4] g/d in estimated 24-hour UNa, Table S1) between the lowest and highest quintiles. The highest quintile of UNa excretion had a higher percentage of participants that were male, non-White, current smokers, with lower education, and higher BMI.

**Table.**
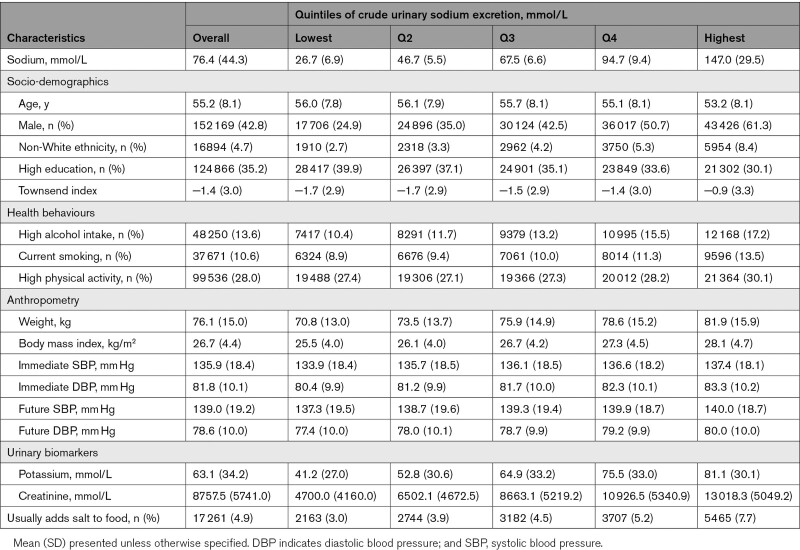
Characteristics of UK Biobank Participants Overall and by Quintile of Crude Urinary Sodium Excretion (mmol/L)

Estimates of within-person variability between baseline and resurvey UNa were extreme, with self-correlations (ρ) of 0.35 for crude UNa and, after adjustment for covariates in the estimating equation, 0.32 for the INTERSALT 24-hour UNa (g/d; ρ=0.80 before adjustment for covariates). For covariates, estimates of within-person variability were low for BMI (ρ=0.92), but moderate for physical activity (0.55) and high for urinary potassium (0.26; Table S2). There was a strong confounding effect of sex, but since heterogeneity by sex was neither significant nor substantive, the sex-specific associations are presented in Table S3 and Figure S4.

### Cross-Sectional Associations

**Figure 1.**
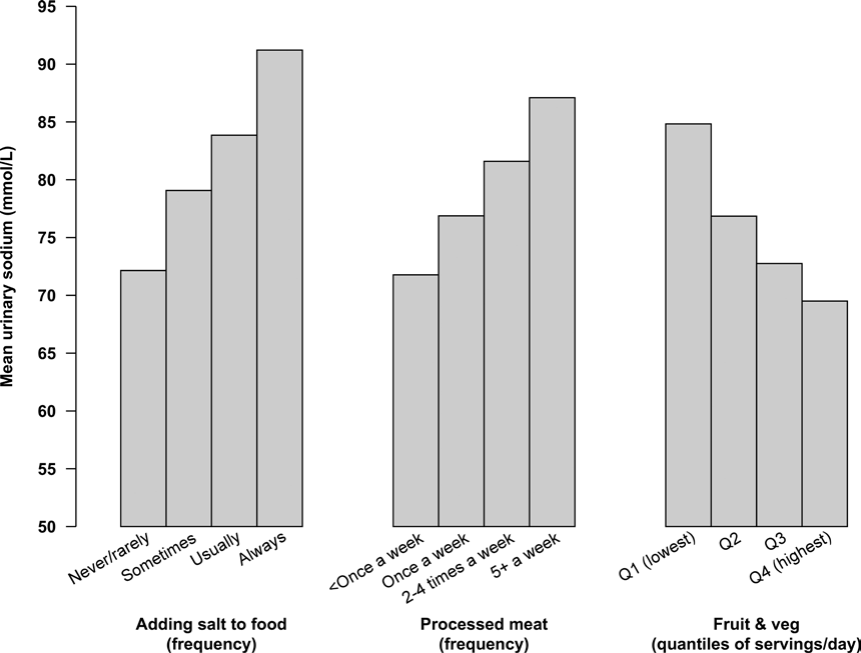
Mean levels of urinary sodium (mmol/L) by levels of self-reported dietary intake (adjusted for age and sex; N=355 134).

Analyses of age- and sex-adjusted crude UNa levels with self-reported dietary intake demonstrated linear associations between higher levels of UNa with higher intakes of processed meat or added salt, and an inverse association with fruit and vegetable consumption (Figure 1). However, despite the apparent associations between these markers of sodium/potassium intake and UNa, correlations between the variables were low (ρ=0.12 [added salt], ρ=0.18 [processed meat], and ρ=−0.19 [fruit/veg]).

**Figure 2.**
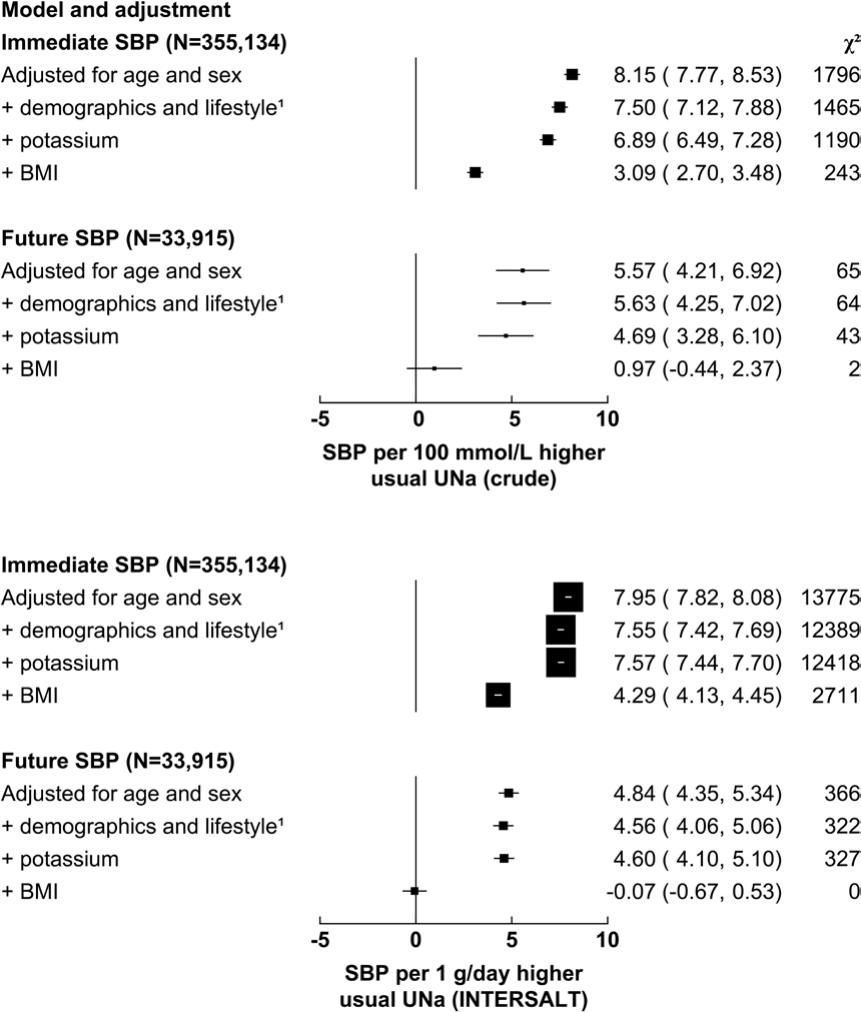
**Associations of usual urinary sodium (UNa) with immediate (N=355 134) and future systolic blood pressure (SBP; N=33 915) before and after adjustment for confounding factors.** + ethnicity, family history, time of sample, season of sample, fluids, education, Townsend index, smoking, alcohol intake, physical activity, and hormone replacement therapy. BMI indicates body mass index.

**Figure 3.**
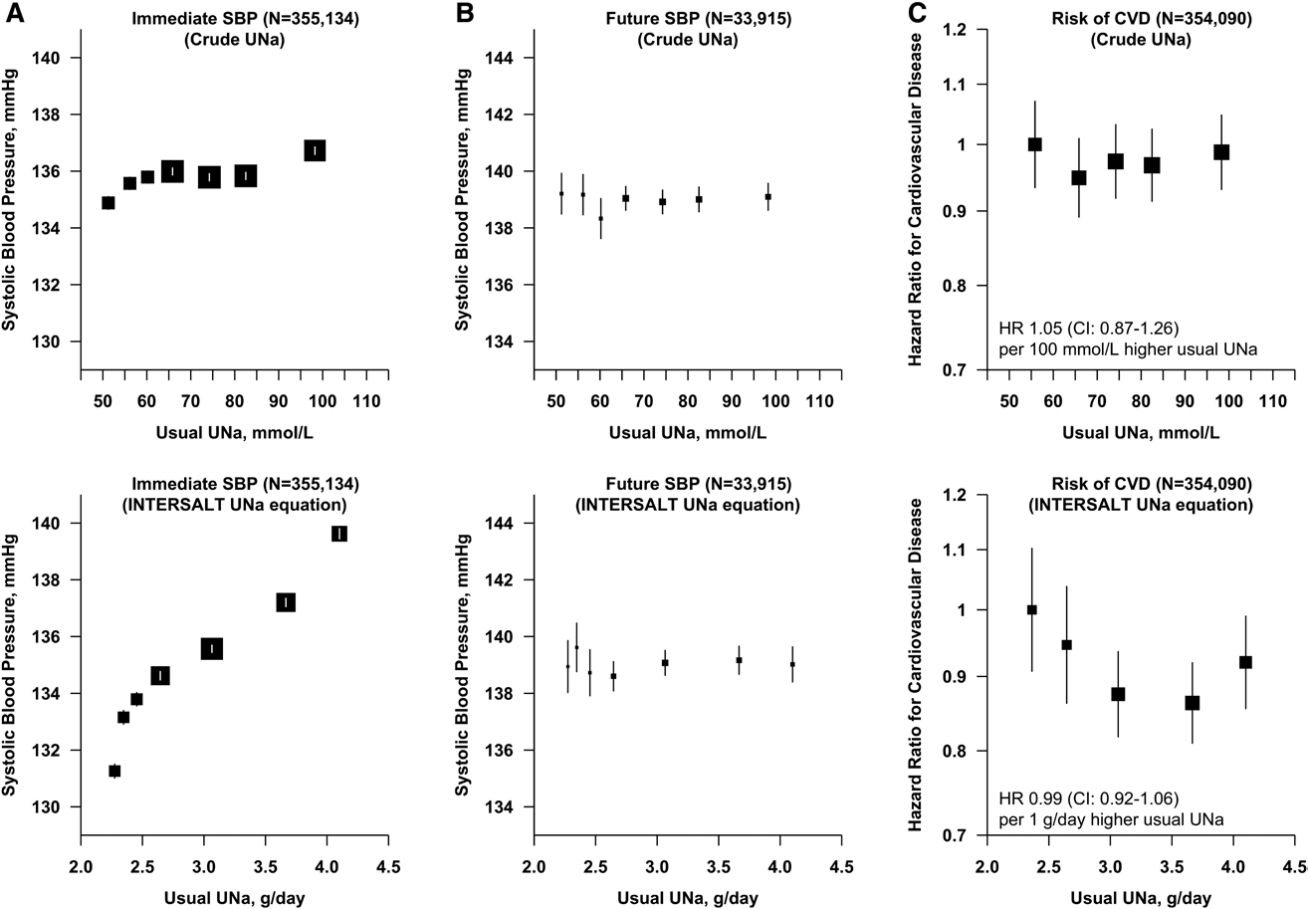
**Associations of usual urinary sodium (UNa) with immediate systolic blood pressure (SBP), future SBP, and risk of cardiovascular disease (CVD) after adjustment for confounding factors (as per Figure 2).** HR indicates hazard ratio.

In baseline models adjusted for age and sex, mean levels of immediate SBP increased by an average of 8.2 mm Hg (95% CI, 7.8–8.5, *P*<0.001) for each 100 mmol/L higher usual UNa (Figure 2). Sequential adjustment for confounders showed that adjustment for sociodemographic and health-related behaviours (model 2) accounted for 18% of the association (χ^2^: 1796 versus 1465). Adjustment for potassium marginally attenuated the association, but adjustment for BMI explained 80% of the remaining association between UNa and SBP (χ^2^: 1190 versus 243). After full adjustment, immediate SBP was an average of 3.1 (95% CI, 2.7–3.5, *P*<0.001) mm Hg higher for each 100 mmol/L higher usual UNa, with a linear positive association across the range of UNa values studied (Figure 3A). The association with immediate SBP was slightly weaker, but broadly equivalent, when the sample was restricted to the subset of participants with data on SBP from both surveys (N=33 915; Figure S3).

### Prospective Analyses

The associations of baseline UNa with future SBP measured an average of 9 years later were only half as strong in the minimally adjusted models for age and sex compared with those in the cross-sectional analyses (χ^2^: 65 versus 124; Figure S3) and were no longer significant after full adjustment for covariates (0.97 mm Hg [95% CI, −0.44 to 2.40], *P*=0.18; Figure 2; Figure 3B). Despite a stronger positive linear association between each 1 g/d higher 24-hour usual UNa (INTERSALT) and immediate SBP than was reported for crude UNa and immediate SBP (4.3 versus 3.1 mm Hg), there was likewise no remaining association between 24-hour UNa (INTERSALT) and future SBP (−0.07 mm Hg [−0.7 to 0.5]; Figure 2; Figure 3B).

The analysis of baseline UNa with incident CVD involved 5566 events occurring during an average of 7.9 years of follow-up. There was no association between a 100 mmol/L higher usual UNa excretion with risk of CVD (HR, 1.05 [95% CI, 0.87−1.26], *P*=0.55, Figure 3C), nor with 24-hour usual UNa (INTERSALT, HR, 0.99 [95% CI, 0.92−1.06], *P*=0.76).

Lastly, sensitivity analyses confirmed that the association of SBP with risk of CVD in this cohort was linear and positive as expected (Figure S5).

## Discussion

The present study demonstrated linear positive associations of higher levels of UNa excretion with immediate SBP but not between baseline UNa values and future SBP recorded at 9 years after baseline. Since SBP is the chief mechanism through which UNa is likely to affect CVD, the lack of association between baseline UNa and future SBP suggested that no association was likely to be detected with incident CVD over the corresponding follow-up period, as observed in the present analyses.^22^

Several hypotheses have been suggested to explain the discrepant results between the beneficial effects observed in randomized trials of sodium restriction on blood pressure, and the higher risks of CVD associated with low sodium intake in prospective studies. The present study demonstrated positive associations between UNa and immediate blood pressure but no association between sodium and subsequent SBP or incident CVD. One potential explanation is that there is an adverse effect of low sodium intake on CVD, which may counteract any beneficial effect of low UNa on SBP, such as the activation of the renin-angiotensin-aldosterone system.^10^ However, one meta-analysis reported no effects of sodium restriction on catecholamine levels,^5^ and another study reported only modest increases in plasma renin activity, aldosterone, and noradrenaline that would likely attenuate over a longer duration of follow-up.^28,29^

An alternative explanation for the discrepant effects reported between the randomized trials and observational studies is the contrasting methods used for measurement of sodium intake. Randomized trials, which typically involve a much smaller number of participants, frequently use gold standard multiple 24-hour urine collections to estimate UNa and hence sodium intake.^30^ However, use of such methods is not feasible in large observational studies, where it is only feasible to collect single spot or timed urine collections (which are then used to estimate 24-hour UNa using algorithms with spot creatinine excretion). While some validation studies have downplayed biases inherent in spot measurements, the impact of validation estimates in the range of 0.30 to 0.50 cannot be underestimated.^31^ Previous validation studies have reported that >50% of individuals would be misclassified when ranking participants by estimates of 24-hour UNa.^17,32–34^ One study demonstrated the effect of this error by comparing associations with incident CVD and mortality from a single baseline measurement of 24-hour sodium versus the average of multiple 24-hour sodium measurements over 5 years.^35^ No association was documented when a single measurement was used, but the use of multiple 24-hour measurements demonstrated that over 50% of participants moved tertiles of sodium intake, and positive associations with incident disease were up to 85% stronger.^35^ In the present study, correlations between spot UNa measurements and self-reported intake of foods high in sodium were low, suggesting low convergent validity between rankings of participants. The potential impact of such substantial measurement error in spot UNa measurements was demonstrated in the present study as the associations of baseline UNa with immediate SBP were completely attenuated when associations with repeat measurements of SBP were examined at a resurvey 9 years after baseline. If the strong, positive, and linear associations with blood pressure (as consistently demonstrated by randomized trials)^28^ are obscured by high levels of within-person variability in spot UNa measurements, it is unlikely that any risk of CVD could be detected over the corresponding follow-up period.

Results of observational studies of UNa also need to consider the potential impact of using algorithms to estimate 24-hour UNa from single spot urine measurements. The most widely used estimation equations rely on the assumption that the ratio of spot sodium to spot creatinine is equivalent over a 24-hour period.^14,36^ First, the assumption of a constant ratio between spot sodium and creatinine and 24-hour measurements is problematic, especially as spot creatinine excretion can be influenced by confounders such as recent protein intake and exercise.^14,36^ Second, the incorporation of age, sex and BMI into the estimation of UNa may result in spurious correlations since each of these variables has their own relationship with both sodium intake and CVD.^37^ This may explain why, in the present study, the association of estimated 24-hour UNa excretion with the INTERSALT equation and mean levels of SBP at baseline was much stronger than those using crude UNa values. However, analyses of the reproducibility of both INTERSALT and crude UNa were concordant after inclusion of the covariates in the estimating equation. Hence, despite the stronger associations reported between estimated 24-hour UNa excretion (INTERSALT) and immediate SBP, associations between baseline 24-hour UNa and future SBP were still completely attenuated, and there was no overall association with incident CVD.

Lastly, observational studies assessing associations between low sodium intake and CVD risk should also consider the possible effects of confounding factors arising from the comparison of different populations using an ecological study design or reverse causality in individuals at high-risk of CVD, where subclinical or prevalent disease leads to a change in either sodium intake or excretion.^29,38^ The large international PURE Study (Prospective Urban Rural Epidemiological) cohort was constrained by both limitations, which could account for their reported J-shaped associations between sodium excretion and CVD.^10^ In contrast, recent prospective analyses of UNa and risk of CVD in UK Biobank, which included more rigorous control for prior disease and studied a more homogenous population, found no association between low UNa and higher risks of CVD.^16,39^

### Strengths and Limitations

The present study involving a large population-based cohort provided a reliable assessment of the shape of the association of UNa excretion with SBP and with risk of CVD, even when the lowest quintile of sodium was further divided into tertiles. Strategies to reduce the effects of reverse causality bias involved excluding participants whose sodium intake or excretion may have been affected by a diagnosis of disease, such as those with self-reported CVD events before baseline assessment or use of antihypertensive medication. However, antihypertensive medication may also be on the causal pathway relating sodium intake to SBP, so this restriction may have attenuated the reported associations. Alternatively, including such participants in the analysis could have biased the associations depending on how such medication affected sodium excretion, or if participants had changed their sodium intake as part of dietary advice following a diagnosis of hypertension. A wide range of variables that may have confounded these analyses were adjusted for, but there may still be residual confounding due to unmeasured confounders (eg, total energy intake) and in self-reported confounders which are prone to reporting biases (eg, physical activity, smoking, and medications). Considering the analysis of within-person variability, adjustment for confounders measured with much higher reproducibility than sodium, such as BMI, may have contributed to why this variable explained so much of the association in Figure 2. Conversely, adjustment for confounders with lower reproducibility could have led to further residual confounding as a result of not adjusting for their usual values. As such, the associations reported in this study are not considered to be causal. Lastly, the study corrected for regression dilution bias arising from random error, but validation studies have also reported potential systematic errors in spot UNa measurements, which could bias associations with disease in any direction.^17,33^

### Perspectives

Prospective observational studies of UNa and risk of CVD have been used to challenge public health advice on restriction of salt intake to lower SBP and prevent CVD. While the limitations of measurements of dietary sodium intake have been widely reported, few studies have appreciated the magnitude of both measurement error and within-person variability in spot measurements of UNa, and their impact on associations with CVD outcomes. The present study demonstrated that the magnitude of within-person variability of spot UNa was so extreme that it completely attenuated any potential prospective associations with blood pressure. In contrast, meta-analyses of randomized trials have consistently demonstrated that sodium restriction lowers blood pressure.^40^ Despite some claims of a potential J-shaped association of CVD at lower levels of sodium intake, the recent 2019 review of Dietary Reference Intake Values for sodium by the National Academies of Sciences, Engineering, and Medicine in the United States and Canada has continued to advocate that adults reduce their sodium intake to 2.3 g/d based on worldwide evidence on this topic, particularly from evidence of randomized trials.^41^ It is important to recognize the limitations of observational studies, irrespective of their study size, and the importance of within-person variability in prospective studies. Failure to account for such biases can obscure true associations of dietary salt restriction on levels of blood pressure and the risk of CVD. Public health recommendations need to be guided by randomized trials of sodium restriction rather than relying on observational studies to address such questions.

## Sources of Funding

This work was supported by core grants to CTSU (Clinical Trial Service Unit) from the Medical Research Council (Clinical Trial Service Unit A310) and the British Heart Foundation (CH/1996001/9454), and by the National Institute for Health Research Oxford Biomedical Research Centre (J.L. Carter and S. Lewington).

## Disclosures

M. Arnold was affiliated with University of Cambridge when the primary work on this publication was completed, but he is now an employee of AstraZenca. The other authors report no conflicts.

## Supplementary Material



## Novelty and Significance

### What Is New?

- The within-person variability of spot urinary sodium measurements in the large prospective UK Biobank is extreme.
- There was a linear positive association between urinary sodium and immediate systolic blood pressure, but no association between sodium and prospective blood pressure or incident cardiovascular disease.

### What Is Relevant?

- Spot measurements of urinary sodium excretion were positively associated with immediate effects on systolic blood pressure, but the magnitude of within-person variability in urinary sodium excretion was so extreme as to preclude any possibility of detecting associations with future systolic blood pressure or incident cardiovascular disease.

### Summary

Failure to appreciate the effects of within-person variability in prospective cohort studies, irrespective of the study size, can lead to erroneous claims about hazards or a lack of benefit.
