# Supplementary figures and images for: Effects of Within-Person Variability in Spot Urinary Sodium Measurements on Associations With Blood Pressure and Cardiovascular Disease
Source: Hypertension. 2021 Sep 20;78(5):1628–36. doi: 10.1161/HYPERTENSIONAHA.120.16549 (PMC7611839; doi:10.1161/HYPERTENSIONAHA.120.16549)

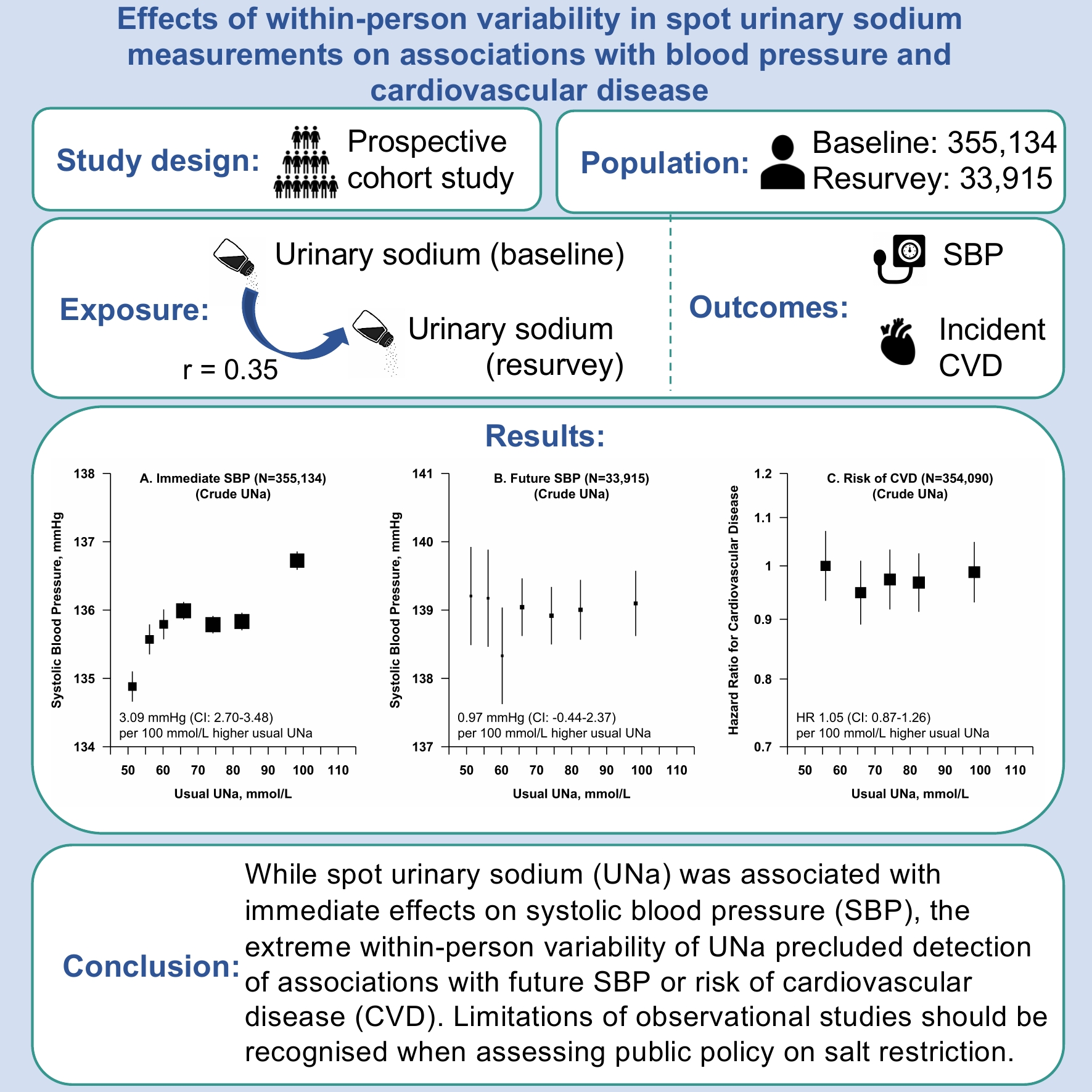

Supplement: Supplementary file 1 [file hyp-78-1628-s001.jpg]
