# Supplementary material for: Effects of Within-Person Variability in Spot Urinary Sodium Measurements on Associations With Blood Pressure and Cardiovascular Disease
Source: Hypertension. 2021 Sep 20;78(5):1628–36. doi: 10.1161/HYPERTENSIONAHA.120.16549 (PMC7611839; doi:10.1161/HYPERTENSIONAHA.120.16549)
Supplement: Supplementary file 2 [file hyp-78-1628-s002.docx]

**DATA SUPPLEMENT**

# Effects of within-person variability in spot urinary sodium measurements on associations with blood pressure and cardiovascular disease

Federica RE MSc^1^, Imen HAMMAMI PhD^1^, Thomas J. LITTLEJOHNS PhD^1^, Matthew ARNOLD PhD^2^, Sarah LEWINGTON^†^ DPhil^1,3^, Robert J. CLARKE^†^ FRCP^1^, Jennifer L. CARTER^†^ PhD^1^

^1^Clinical Trial Service Unit and Epidemiological Studies Unit (CTSU), Nuffield Department of Population Medicine, University of Oxford, Oxford, UK.

^2^ British Heart Foundation Cardiovascular Epidemiology Unit, Department of Public Health and Primary Care, University of Cambridge, Cambridge, UK, CB1 8RN

^3^ MRC Population Health Research Unit

^†^ Joint Senior Authorship

**Address for correspondence**

Dr Jennifer Carter

CTSU, Nuffield Department of Population Health

University of Oxford
Big Data Institute

Old Road Campus

Oxford, OX3 7LF, UK

Tel: 44-1865-743743

Fax: 44-1865-743985

Email: [jennifer.carter@ndph.ox.ac.uk](mailto:jennifer.carter@ndph.ox.ac.uk)

Table of Contents

[Effects of within-person variability in spot urinary sodium measurements on associations with blood pressure and cardiovascular disease 1](#_Toc78965590)

[Supplemental Methods S1. Formulae for estimating 24-hr sodium excretion (g/day). 3](#_Toc78965591)

[Supplemental Methods S2. Additional details on covariate coding and adjustment. 4](#_Toc78965592)

[Supplemental Table S1. Characteristics of UK Biobank participants overall and by quintile (Q) of estimated 24-hr sodium excretion (g/day) with the INTERSALT and Kawasaki equations. 5](#_Toc78965593)

[Supplemental Table S2. Self-correlation of covariates included in analyses of urinary sodium excretion 7](#_Toc78965594)

[Supplemental Table S3. Sex-specific characteristics of UK Biobank participants overall and by quintile (Q) of crude urinary sodium excretion (mmol/L). 8](#_Toc78965595)

[Supplemental Figure S1. Flowchart of UK Biobank surveys 10](#_Toc78965596)

[Supplementary Figure S2. 11](#_Toc78965597)

[Supplemental Figure S3. Associations of usual baseline sodium (UNa) with immediate and future systolic blood pressure (SBP) when restricting all analyses to the subsample with future SBP (N=33,915). 12](#_Toc78965598)

[Supplementary Figure S4. Sex-specific associations of urinary sodium with immediate and future systolic blood pressure (SBP) before and after adjustment for confounding factors. 13](#_Toc78965599)

[Supplemental Figure S5. Cox regression of the fully adjusted^1^ association between systolic blood pressure (SBP) at baseline and the risk of cardiovascular disease (CVD) in UK Biobank 14](#_Toc78965600)

Supplemental Methods S1. Formulae for estimating 24-hr sodium excretion (g/day).

INTERSALT Equation (incorporating the constant for Western Europe): ^1^

Male =23*((25.46+(0.46*sodium[mmol/L])+(-2.75*creatinine[mmol/L])+(-0.13*potassium[mmol/L])+(4.10*bmi)+(0.26*age)+17.05))

Female=23*((5.07+(0.34*sodium[mmol/L)+(-2.16*creatinine[mmol/L])+(-0.09*potassium[mmol/L))+(2.39*bmi)+(2.35*age)+

(-0.03*age^2^)+12.82))

Kawasaki Equation:^2^

24-hr UNa (g/day) =(23 * [16.3 * XNa^0.5^])/1000, where:

XNa = (spot Na [mmol/l] / [spot Creatinine (mg/dL) * 10]) *Predicted_24hr_creatinine (“Pr24hrCr”;mg/day)

Male: Pr24hrCr (mg/day) = (-12.63 * age [year]) + (15.12 * weight [kg]) + (7.39 * height [cm]) -79.9

Female: Pr24hrCr (mg/day) = (-4.72 * age [year]) + (8.58 * weight [kg]) + (5.09 * height [cm])-74.5

^1^ Brown I, Dyer A, et al. Estimating 24-hour urinary sodium excretion from casual urinary sodium concentrations in Western populations: The INTERSALT Study. *American Journal of Epidemiology*. 2013;177(11):1180-1192.

^2^ Kawasaki T, Itoh K, Uezono K, Sasaki H. A simple method for estimating 24H urinary sodium and potassium excretion from second morning voiding urine specimen in adults. *Clinical and Experimental Pharmacology and Physiology*. 1993;20(I):7-14.

# Supplemental Methods S2. Additional details on covariate coding and adjustment.

The baseline Model 1 adjusted for age and sex only. Model 2 included additional adjustment for sociodemographic factors and health behaviours (ethnicity [1=Non-White]; family history of hypertension [1=yes]; season; time of day of urine sample; usual fluid consumption [ml/day from tea, coffee and water intake]; education [none, O/A levels, professional qualifications, degree+]; Townsend index of deprivation [continuous, based on participants’ postcode at recruitment,^1^]; smoking [never, ex-smoker, current]; alcohol intake [none, low (<14 units/week), moderate (14-35 units/wk), high (>35 units/wk)], physical activity [<10 metabolic equivalent of task (MET) hr/week, 10-50 MET hr/week, >=50 MET hr/week from the Physical Activity Questionnaire^2^; and exogenous hormones [1=yes]). Model 3 included additional adjustment for the spot urinary potassium/creatinine ratio.^3^ Finally, Model 4 also adjusted for BMI. The same adjustments were also used when assessing the associations between estimated 24-hr UNa (using INTERSALT equation), SBP, and CVD in multivariable models (Supplemental Figures S2).

^1^ Townsend P, Beattie A, Phillimore P. Health and Deprivation: Inequality and the North. London, UK: Routledge; 1988.

^2^ Cassidy S, Chau J, Catt M, Bauman A, Trenell M. Cross-sectional study of diet, physical activity, television viewing and sleep duration in 233,110 adults from the UK Biobank; the behavioural phenotype of cardiovascular disease and type 2 Diabetes. *BMJ Open*. 2016;6: e010038. doi:10.1136/bmjopen-2015-010038.

^3^Jędrusik P, Symonides B, Wojciechowska E, Gryglas A, Gaciong Z. Diagnostic value of potassium level in a spot urine sample as an index of 24-hour urinary potassium excretion in unselected patients hospitalized in a hypertension unit. Shimosawa T, ed. *PLOS ONE*. 2017;12(6). doi:10.1371/journal.pone.0180117

Supplemental Table S1. Characteristics of UK Biobank participants overall and by quintile (Q) of estimated 24-hr sodium excretion (g/day) with the INTERSALT and Kawasaki equations. Mean (SD) presented unless otherwise specified.

| **Characteristics** | **Overall** | **INTERSALT Equation** | |  | **Kawasaki Equation** | |
| --- | --- | --- | --- | --- | --- | --- |
|  |  | Lowest Q1 | Highest Q5 |  | Lowest Q1 | Highest Q5 |
| Sodium, mmol/l | 76.4 (44.3) | 49.0 (25.9) | 114.6 (49.2) |  | 48.7 (26.4) | 105.7 (51.8) |
| Sodium, g/day | 3.2 (0.8) | 2.3 (0.2) | 4.4 (0.4) |  | 2.6 (0.4) | 5.7 (0.7) |
| **Socio-demographics** |  |  |  |  |  |  |
| Age, years | 55.2 (8.1) | 59.5 (7.3) | 55.0 (8.2) |  | 56.6 (8.0) | 53.4 (8.0) |
| Male, N (%) | 152169 (42.8) | 2690 (3.8) | 68771 (96.8) |  | 17580 (24.8) | 45902 (64.6) |
| Non-white ethnicity, N(%) | 16894 (4.7) | 1883 (2.7) | 4182 (5.9) |  | 2780 (3.9) | 4397 (6.2) |
| High education, N(%) | 124866 (35.2) | 24821 (34.9) | 21164 (29.8) |  | 25885 (36.4) | 23282 (32.8) |
| Townsend index, most  deprived quintile, N(%) | 71024 (20.0) | 12220 (17.2) | 15882 (22.4) |  | 13729 (19.3) | 16163 (22.8) |
| **Health behaviours** |  |  |  |  |  |  |
| High alcohol intake, N(%) | 48250 (13.6) | 4889 (6.9) | 16738 (23.6) |  | 7810 (11.0) | 12618 (17.8) |
| Current smoking, N(%) | 37671 (10.6) | 5924 (8.3) | 8967 (12.6) |  | 7034 (9.9) | 8964 (12.6) |
| High exercise, MET hrs, N(%) | 99536 (28.0) | 20286 (28.6) | 22381 (31.5) |  | 18714 (26.3) | 22154 (31.2) |
| **Anthropometry** |  |  |  |  |  |  |
| Body mass index, kg/m^2^ | 26.7 (4.4) | 24.0 (3.3) | 29.6 (4.3) |  | 26.0 (4.3) | 27.8 (4.7) |
| Immediate SBP, mmHg | 135.9 (18.4) | 133.4 (19.1) | 142.8 (17.2) |  | 132.5 (18.2) | 139.6 (18.2) |
| Immediate DBP, mmHg | 81.8 (10.1) | 78.4 (9.6) | 86.3 (9.7) |  | 79.8 (10.0) | 84.3 (10.1) |
| Future SBP, mmHg | 139.0 (19.2) | 137.1 (20.3) | 144.6 (17.6) |  | 137.8 (19.6) | 140.1 (18.5) |
| Future DBP, mmHg | 78.6 (10.0) | 75.5 (10.0) | 81.7 (9.6) |  | 77.5 (10.0) | 80.0 (10.0) |
| **Urinary biomarkers** |  |  |  |  |  |  |
| Potassium, mmol/l | 63.1 (34.2) | 70.4 (39.2) | 63.2 (28.8) |  | 79.0 (38.4) | 51.8 (27.1) |
| Creatinine, mmol/l | 8757.5 (5741.0) | 8923.8 (6927.5) | 10045.5 (4688.0) |  | 11919.3 (7124.9) | 6669.9 (3995.3) |
| Usually adds salt to food, N(%) | 17261 (4.9) | 2481 (3.5) | 4763 (6.7) |  | 2639 (3.7) | 4807 (6.8) |

SBP= systolic blood pressure; DBP=diastolic blood pressure; MET= metabolic equivalents

Supplemental Table S2. Self-correlation of covariates included in analyses of urinary sodium excretion**.**

| **Covariate** | **Self-correlation**  **(Spearman’s Rho)** |
| --- | --- |
| Weight | 0.95 |
| Height | 0.99 |
| BMI | 0.92 |
| Potassium | 0.26 |
| Creatinine | 0.38 |
| Alcohol intake | 0.86 |
| Physical activity | 0.55 |
| Fluid intake | 0.66 |
| Smoking status^a^ | 0.87 |

^a^ Kappa statistic

Supplemental Table S3. Sex-specific characteristics of UK Biobank participants overall and by quintile (Q) of crude urinary sodium excretion (mmol/L). Mean (SD) presented unless otherwise specified.

|  | **FEMALE** | | |  | **MALE** | | |
| --- | --- | --- | --- | --- | --- | --- | --- |
| **Characteristics** | **Overall** | **Lowest Q1** | **Highest Q5** |  | **Overall** | **Lowest Q1** | **Highest Q5** |
| Sodium, mmol/l | 66.8 (40.3) | 26.3 (6.9) | 144.4 (28.2) |  | 89.3 (46.2) | 27.7 (6.6) | 148.6 (30.2) |
| **Socio-demographics** |  |  |  |  |  |  |  |
| Age, years | 55.3 (8.0) | 56.1 (7.7) | 52.8 (8.1) |  | 55.1 (8.2) | 55.9 (8.1) | 53.4 (8.2) |
| Non-white ethnicity, N(%) | 9456 (4.7) | 1404 (2.6) | 2624 (9.6) |  | 7438 (4.9) | 506 (2.9) | 3330 (7.7) |
| High Education, N(%) | 68552 (33.8) | 20577 (38.5) | 7871 (28.7) |  | 56314 (37.0) | 7840 (44.3) | 13431 (30.9) |
| Townsend index, most  deprived quintile, N(%) | 39840 (19.6) | 9045 (16.9) | 7384 (26.9) |  | 29989 (20.5) | 3345 (18.9) | 10746 (24.8) |
| **Health behaviours** |  |  |  |  |  |  |  |
| High alcohol intake, N(%) | 12757 (6.3) | 3247 (6.1) | 1957 (7.1) |  | 35493 (23.3) | 4170 (23.6) | 10211 (23.5) |
| Current smoking, N(%) | 18221 (9.0) | 4114 (7.7) | 3234 (11.8) |  | 19450 (12.8) | 2210 (12.5) | 6362 (14.7) |
| High exercise, MET hrs,  N(%) | 52517 (25.9) | 14159 (26.5) | 6933 (25.3) |  | 47019 (30.9) | 5329 (30.1) | 14431 (33.2) |
| **Anthropometry** |  |  |  |  |  |  |  |
| Weight, kg | 69.9 (13.1) | 67.4 (11.5) | 73.9 (15.1) |  | 84.4 (13.3) | 80.8 (12.0) | 87.0 (14.3) |
| Body mass index, kg/m^2^ | 26.4 (4.7) | 25.3 (4.1) | 28.0 (5.5) |  | 27.2 (3.8) | 26.0 (3.4) | 28.1 (4.1) |
| Immediate SBP, mmHg | 133.2 (18.8) | 132.4 (18.6) | 133.3 (19.0) |  | 139.6 (17.1) | 138.2 (17.1) | 140.0 (17.0) |
| Immediate DBP, mmHg | 80.1 (9.9) | 79.5 (9.7) | 81.0 (10.2) |  | 84.0 (9.9) | 83.1 (9.9) | 84.8 (9.9) |
| Future SBP, mmHg | 135.7 (19.6) | 135.2 (19.6) | 135.4 (19.6) |  | 142.8 (18.1) | 142.5 (18.5) | 142.5 (17.6) |
| Future DBP, mmHg | 76.8 (9.9) | 76.3 (9.9) | 77.7 (9.9) |  | 80.7 (9.8) | 80.2 (9.7) | 81.3 (9.8) |
| **Urinary biomarkers** |  |  |  |  |  |  |  |
| Potassium, mmol/l | 58.9 (33.4) | 40.6 (26.3) | 79.4 (30.2) |  | 68.7 (34.3) | 43.0 (29.1) | 82.2 (29.9) |
| Creatinine, mmol/l | 7112.9 (4870.2) | 4248.2 (3580.7) | 11247.7 (4473.5) |  | 10951.0 (6071.6) | 6065.2 (5326.9) | 14137.8 (5072.1) |
| Usually adds salt to food, N(%) | 9296 (4.6) | 1534 (2.9) | 2201 (8.0) |  | 7965 (5.2) | 629 (3.6) | 3264 (7.5) |

SBP= systolic blood pressure; DBP=diastolic blood pressure; MET= metabolic equivalents

Supplemental Figure S1. Flowchart of UK Biobank surveys relevant to the analysis in this study on urinary sodium excretion, systolic blood pressure (SBP) and cardiovascular disease (CVD).


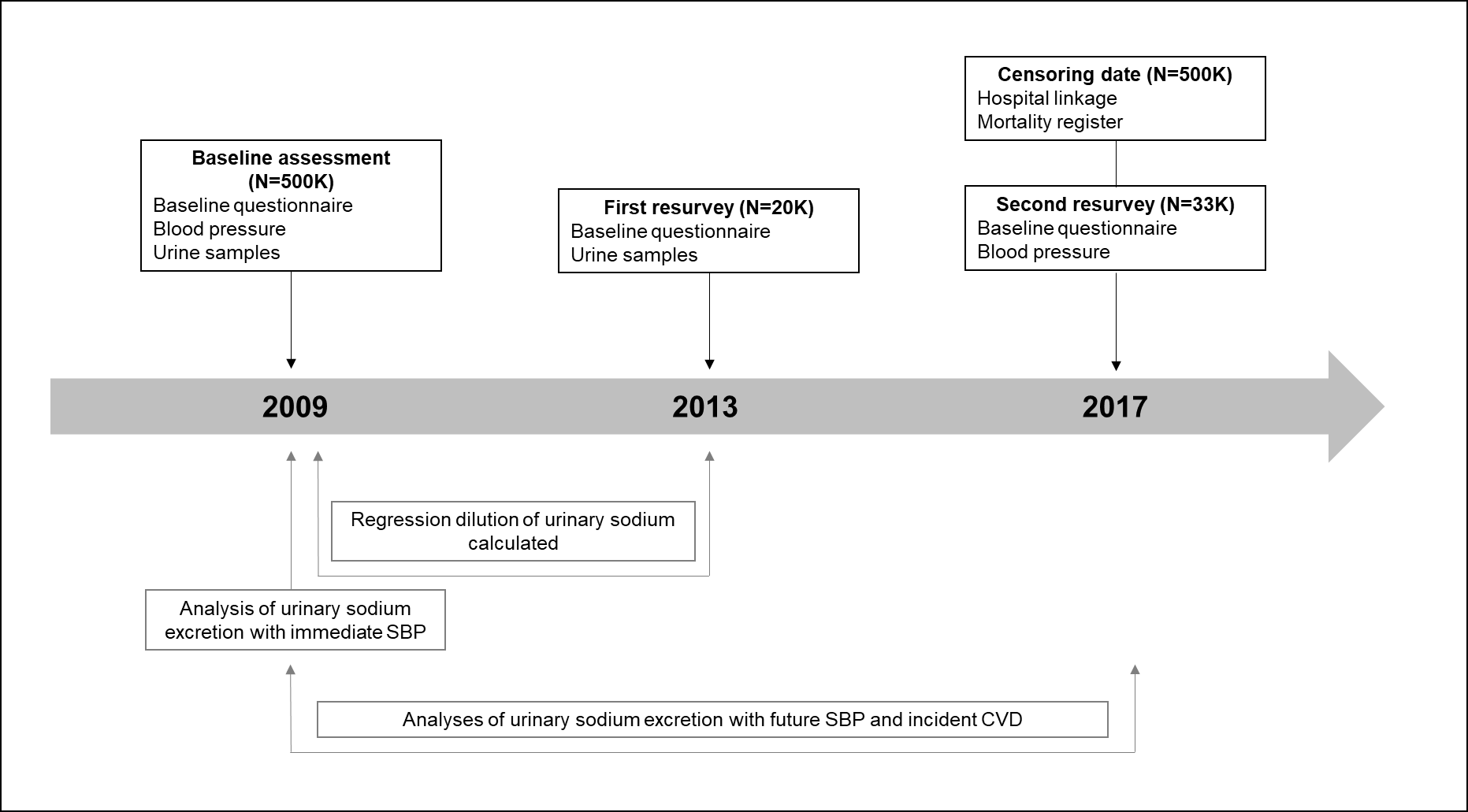


Supplementary Figure S2. Fully adjusted associations of systolic blood pressure (SBP) and incident cardiovascular disease (CVD) with estimated 24-hr usual sodium excretion (1 g/day) from the Kawasaki equation.

# **Supplemental Figure S3.** Associations of usual baseline sodium (UNa) with immediate and future systolic blood pressure (SBP) when restricting all analyses to the subsample with future SBP (N=33,915).


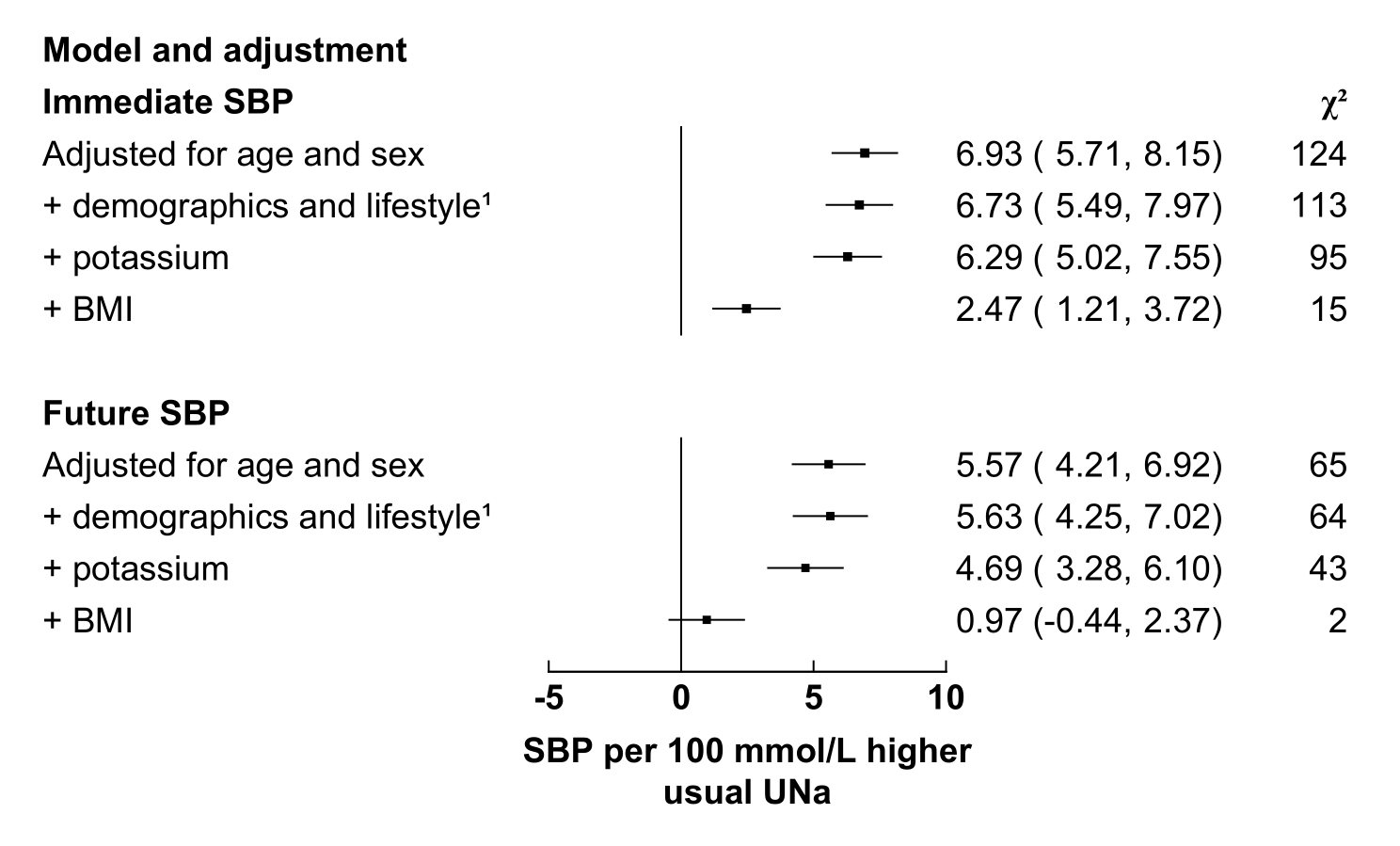


# **Supplementary Figure S4**. Sex-specific associations of urinary sodium with immediate and future systolic blood pressure (SBP) before and after adjustment for confounding factors.





^1^ + ethnicity, family history, time of sample, season of sample, fluids, education, Townsend index, smoking, alcohol intake, exercise and hormone replacement therapy.

Supplemental Figure S5. Cox regression of the fully adjusted^1^ association between systolic blood pressure (SBP) at baseline and the risk of cardiovascular disease (CVD) in UK Biobank (N=420,682).


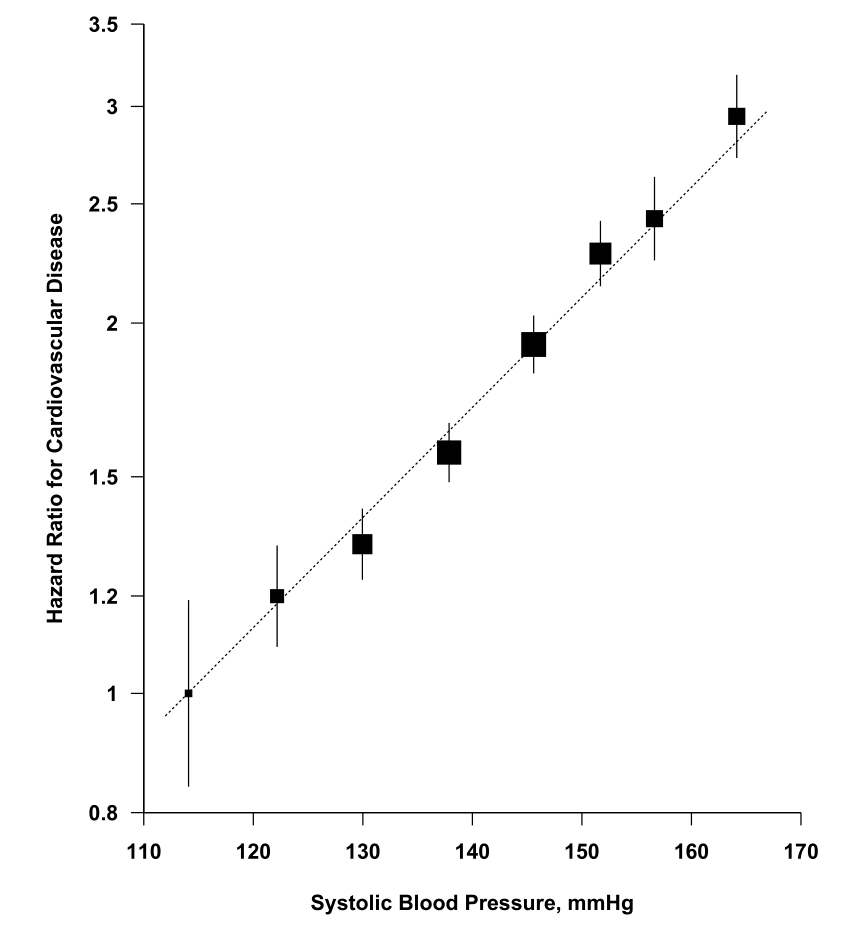


^1^ Association is adjusted for sex, age, ethnicity, education, townsend index, smoking status, alcohol, exercise, BMI. Participants on hypertensive medication are not excluded (as they are in the main analyses of sodium, SBP and CVD). Cox regression is stratified by five year age groups.
